# Current state of training needs and programs for infection control liaison nurses

**DOI:** 10.3389/fmed.2025.1703523

**Published:** 2025-12-17

**Authors:** Li Ni, Qingqing Du, Hailei Bian, Norhafizah Ab Manan, Abd Rahim Mohamad

**Affiliations:** 1 University of Cyberjaya, Cyberjaya, Selangor, Malaysia; 2 Shanghai East Hospital, School of Medicine, Tongji University, Shanghai, China; 3 Shanghai Children's Hospital, School of Medicine, Shanghai Jiao Tong University, Shanghai, China; 4 Tongji University School of Medicine, Shanghai, China

**Keywords:** infection control liaison nurse, training model, training needs, training program, job competency

## Abstract

Amid the continuous advancement of medical technologies and increasingly stringent requirements for hospital infection management, the infection control liaison nurse serves as a critical bridge between the infection control department and clinical units. The professional competence and training quality of these nurses directly influence the effectiveness of infection prevention and control measures within hospitals. At present, there is a significant variation in the job competency, mastery of professional knowledge, and application of practical skills among infection control liaison nurses, leading to diversified training needs. The design and implementation of training programs face challenges such as incomplete systems, uniformity in training models, and non-standardized criteria for outcome evaluation. An analysis of the current training landscape reveals significant gaps in the integration of theory and practice, a lack of pertinence in training content, and the absence of a robust mechanism for continuous education. There is an urgent need to develop a more scientific, systematic, and effective training model to enhance the overall quality and professional level of infection control liaison nurses.

## 1 Introduction

The prevention and control of hospital infections is an important part of ensuring patient safety and maintaining medical quality. Infection control liaison nurses (ICLNs), defined as clinical nurses who serve as a bridge between the infection control department and clinical units, are responsible for translating infection control theories into practical actions within their respective units. Unlike full-time infection prevention and control (IPC) practitioners, ICLNs typically maintain clinical responsibilities while assuming additional IPC duties, necessitating a unique set of competencies and targeted training (1). The World Health Organization (WHO) underscores that effective IPC programs require education and training for all health workers, highlighting the systemic importance of equipping specific roles like ICLNs (2). In recent years, with the increasing complexity of the medical environment and the continuous emergence of new infectious diseases, the professional requirements for infection control liaison nurses have also been continuously rising (1). Research shows that there is a significant positive correlation between the psychological empowerment level of infection control personnel and the implementation rate of infection risk indicators. Factors such as self-efficacy, job significance, work autonomy, and work impact directly affect the effectiveness of risk measures (3). Meanwhile, based on the evidence-based clinical infection control pathway, it has shown excellent results in preventing infections. However, this requires infection control personnel to possess higher professional skills and practical abilities (4). The completeness of the training model for infection control liaison nurses is closely related to the overall quality of hospital infection prevention and control work. Therefore, understanding the current situation of their training needs and optimizing the training program hold significant positive implications.

## 2 Methodology

This narrative review synthesized evidence on training needs and programs for infection control liaison nurses (ICLNs). A systematic literature search was conducted using PubMed, Web of Science, CNKI, and Embase for articles published between January 2015 and August 2025. Search terms included: “infection control liaison nurse,” “ICLN,” “infection prevention and control training,” “IPC nurse training,” “competency,” “training needs,” and “training program.”

Inclusion criteria were: (1) studies focusing on ICLNs or part-time infection control nurses; (2) articles addressing training needs, competency, or program evaluation; (3) empirical, review, or guideline documents. Exclusion criteria were: (1) studies not specific to nursing roles; (2) non-English or non-Chinese publications without available translation; (3) conference abstracts without full text.

After screening, 40 studies were included, comprising cross-sectional surveys, intervention studies, qualitative research, and systematic reviews. The synthesis focused on thematic analysis of training needs, program structures, evaluation methods, and international comparisons.

## 3 Analysis of the current status of training needs for infection control liaison nurses

### 3.1 Current status of job competency among infection control personnel

The job competency of infection control personnel in our country shows an overall imbalance in development. There are significant differences between different regions and medical institutions at different levels. Survey research conducted in Zhongshan city reveals that the self-assessed total score of job competency for infection control personnel is 5.37 ± 0.85, (on a 7-point Likert scale, where 1 = very low and 7 = very high), indicating a moderate to high level of self-perceived competency, with scores ranking from highest to lowest in personal characteristics, organizational collaboration abilities, basic infection control skills, and professional development capabilities. Notably, full-time infection control personnel consistently scored higher across all dimensions compared to part-time personnel (5). In the Southwest region, the average score for job competency among hospital infection control personnel was 5.70 ± 0.94, indicating an above-average level overall, yet marked differences persist across hospitals of varying statuses (6). In core dimension evaluations, the highest scores were observed in basic infection control skills, while professional development scored the lowest, underscoring a significant potential for growth in professional expertise among infection control personnel. It is crucial to note that women constitute 83.57% of infection control personnel, with 67.99% having a nursing background, 84.84% holding intermediate or lower professional titles, and 93.91% possessing a bachelor’s degree or lower, highlighting specific characteristics that should guide the design of training programs.

In a cross-sectional survey conducted by She Xiyun et al. (7) involving 117 part-time infection control nurses, encompassing five dimensions, the total job competency score stood at 4.88 ± 0.78, reflecting a moderate level. High levels were observed in infection control knowledge, skills, and implementation abilities, while training and research and evidence-based capabilities were rated as moderate. This suggests that while infection control nurses possess considerable theoretical and practical operational competencies, the conventional theoretical knowledge often fails to meet the needs of daily work, indicating a necessity for ongoing self-improvement in training, research, and evidence-based capabilities to enhance research skills, continually update their knowledge base, and proactively address new challenges in infection control work. Furthermore, a multiple linear regression analysis of factors affecting job competency indicated that the length of service was a significant factor, positively correlated with competency scores. Thus, it is vital to strengthen infection control training for nurses with less than 3 years of service to reduce the competency gap between them and more experienced nurses.

### 3.2 Analysis of training needs

#### 3.2.1 Analysis of the demand for training content

The training needs of infection control liaison nurses are characterized by their multidimensional and multilevel nature, primarily focusing on four core domains: professional competence, comprehensive ability, professionalism, and personal traits, as illustrated in Table 1. Research employing the Analytic Hierarchy Process combined with the Entropy Method to construct a competency evaluation index system reveals that professional competence holds the highest weight (0.362), followed by comprehensive ability (0.317), personal traits (0.197), and professionalism (0.124) (7). Among the secondary indicators, the capability for infection surveillance and risk assessment ranks highest (0.154), succeeded by educational training ability (0.112), professional development (0.101), communication skills (0.101), and research skills (0.088). Specific training content areas include hospital-wide infection control behavior training and supervision, disinfection and environmental monitoring, microbiological monitoring, and standardized use of antibiotics, epidemiological investigation, and risk assessment. Concurrently, with the advancement of medical technology, emerging training needs such as respiratory infectious disease protection, multidrug-resistant organism control, and hand hygiene standards are increasingly emphasized (8, 9). To address these multifaceted needs, the design of training programs must be systematic and stratified, a topic explored in the following section on the current status of training program development.

**Table 1:** Main areas and weight distribution of training needs for infection control liaison nurses (ICLNs).

Primary indicators	Weight	Secondary indicators	Weight	Main training content
Professional competence	0.362	Infection surveillance and risk assessment	0.154	Risk identification, monitoring methods, data analysis
		Basic infection control skills	0.108	Standard precautions, isolation techniques, disinfection, and sterilization
Comprehensive ability	0.317	Educational training ability	0.112	Design of training programs, teaching methods, evaluation of effects
		Communication skills	0.101	Interdepartmental coordination, patient education, team collaboration
		Research skills	0.088	Evidence-based practice, quality improvement, academic writing
Professionalism	0.124	Professional development	0.101	Continuous learning, career planning, professional certification
Personal traits	0.197	Psychological quality	0.089	Stress management, emotional regulation, prevention of professional burnout

#### 3.2.2 Analysis of training modalities and instructor requirements

Concerning training modalities, infection control nurses anticipate training sessions ranging from monthly to quarterly, with each session lasting between 1 and 2 h. The training is to be delivered through a hybrid format, combining both online and face-to-face methods. As for the qualifications of instructors, there is an expectation for educators to possess robust professional competence, extensive experience in both clinical settings and research; furthermore, the instruction should emphasize clarity, structured content, and classroom interaction, while also managing the course duration effectively. Beyond lecture-based instruction, the provision of on-site practical guidance aims to enhance both the practicality and effectiveness of the training (10, 11).

## 4 Current status of the development of the infection control liaison nurse training program

### 4.1 Current state of training model development

Although the construction of the training model for infection control liaison nurses in China is gradually improving, there is still a need for enhancement. The implementation of the ADDIE model, which stands for Analysis, Design, Development, Implementation, and Evaluation, has proven effective in practice for strengthening the capabilities of the infection control team. Post-training, the average quality scores of infection control in medical institutions in Shanghai increased from 92.99 ± 6.07 to 94.46 ± 6.76 (12). This model provides a scientific framework for the systematic development of the training model. Mature training models and certification regimes abroad offer significant insights for China. The World Health Organization (WHO) has outlined core components for effective IPC programmes, which include education and training as fundamental elements, providing a high-level framework for development (2). International evidence consistently highlights the importance of organizational structure and staff roles in effective infection prevention (13). Furthermore, research demonstrates the critical role nurses play in implementing and leading IPC initiatives at the bedside, such as promoting hand hygiene, which underscores the practical competencies required for liaison roles (14). Qualitative studies further help define the specific competencies needed for nurses in infection prevention roles, such as their stake in antimicrobial stewardship, informing the content of training programs (15). The successful application of a three-tiered, multi-dimensional intervention model in primary hospitals demonstrates that a stratified training model can better meet the specific needs of various medical institutions (16). The implementation of a combined infection control team and risk assessment management significantly improved the theoretical knowledge and operational skills of medical personnel; the observation group scored 95.02 ± 4.14 in theoretical knowledge and 96.17 ± 2.67 in practical skills, both markedly higher than the control group (17).

### 4.2 Main modes and methods of training programs

#### 4.2.1 Theoretical training modes and content

Theoretical training serves as a fundamental component in the capacity building of infection control liaison nurses, employing diverse teaching methods and flexible training modalities. Analysis of typical cases of nosocomial infections reveals deficiencies in the clinical thinking related to infection control among clinicians. Records of nosocomial infection risk assessments and interventions accounted for only 40.00%, while the rate of pathogen testing before antimicrobial therapy initiation stood at 43.33%. Furthermore, the issuance rate of isolation orders for contact with multidrug-resistant organisms was merely 15.38% (18). The construction of regionalized infection control platforms has provided a new medium for theoretical training. The city of Shenzhen has significantly enhanced the pathogen testing rate and management efficacy through the advancement of a regionalized hospital infection management platform (19). Additionally, the increasing application of information systems in theoretical training has broadened considerably. The development of infection monitoring information systems within medical institutions has facilitated the standardization of training content and the quantification of training effectiveness assessments, thereby providing technical support (20).

Specifically, in 1982, the Certification Board of Infection Control and Epidemiology in the United States conducted a practical analysis of the duties of infection control professionals to define the responsibilities and training assessment content for these specialists. The training for infection control professionals should encompass the following six categories: (1) identification of infectious processes; (2) monitoring and epidemiological investigation of hospital infections; (3) prevention/control of the transmission of infectious sources; (4) infection management and communication; (5) education and research; and (6) infection control in the health of healthcare personnel. Compared to the systematic training models already established internationally, domestic hospitals in China mainly focus their training content on the key responsibilities and weak points of infection control nurses, self-designed and lacking in systematicity.

#### 4.2.2 Training in practical skills

The training in practical skills constitutes an integral component of the training program for infection control liaison nurses, directly impacting the efficacy of infection control implementations. The bundled approach to nursing care combined with infection control measures has demonstrated significant advantages in preventing ventilator-associated pneumonia. Observational data indicate that the duration of mechanical ventilation and hospital stays are substantially shorter in the intervention group compared to the control group, with a marked reduction in the incidence of ventilator-associated pneumonia (21). The application of a multidimensional, phased nursing intervention model in conjunction with infection control supervision has proven essential in the context of chronic renal failure patients undergoing hemodialysis, underscoring the necessity of tailoring practical skills training to specific clinical scenarios and procedural workflows (22). Moreover, the practice of infection control risk assessment in maternal and child health institutions illustrates that an infection control risk assessment and management model, primarily led by specific departments with support from the hospital infection management division, effectively integrates the unique infection control characteristics of various departments (23).

#### 4.2.3 Enhancement training for comprehensive abilities

The enhancement training for comprehensive abilities aims to develop the overall qualities and capabilities of infection control liaison nurses, encompassing aspects such as communication coordination, educational training, and research innovation. Qualitative research on ICU nurses caring for patients infected with multidrug-resistant organisms reveals the complex emotional experiences and multifaceted needs faced by nurses in practice, including issues related to scheduling and human resource management, learning and training, communication and collaboration, as well as environmental and equipment challenges (24). These findings provide crucial insights for designing the content of comprehensive ability enhancement training for infection control liaison nurses. The application of a grid management strategy in hospital infection management has effectively improved the nursing quality and comprehensive abilities of nursing staff, with the experimental group scoring significantly higher across various dimensions than the control group (25). Furthermore, a three-level infection control strategy combined with multidimensional interventions in the operating rooms of primary hospitals not only enhances patient recovery processes but also boosts the professional qualities and service capabilities of nursing staff (26).

### 4.3 Evaluation system for training effectiveness

#### 4.3.1 Pre-training assessment system

Pre-training assessment serves as a crucial prerequisite to ensure the pertinence and effectiveness of training. It necessitates a comprehensive understanding of the trainees’ foundational level, knowledge structure, and capability deficiencies. A follow-up evaluation study on the application of the “Shaanxi Province ICU Hospital Infection Prevention and Control Standard WS/T509-2016″ revealed that while 94.12% of respondents were familiar with the standard, only 75.29% had undergone training on it (27). This underscores the importance of focusing the pre-training assessment on bridging the gap between theoretical knowledge and practical application, thereby providing a basis for the development of personalized training programs. The application of metagenomic diagnostic techniques in extremely premature infants with congenital tuberculosis and multiple infections indicates that the assessment system should encompass multiple dimensions, including basic knowledge tests, skills assessment, and attitude and behavior surveys, while also considering individual differences and job-specific characteristics (28). Research on the implementation effects of the training program for infection control liaison nurses suggests that a thorough pre-training assessment directly influences the training outcomes (29).

#### 4.3.2 Training process monitoring

Monitoring the training process is a key component in ensuring training quality and effectiveness, requiring the establishment of comprehensive monitoring mechanisms and feedback systems. Basic infection control measures, such as hand hygiene, have been proven effective in reducing hospital-acquired infections (HAIs); however, compliance with these simple measures remains generally low (30). Research under the DRG payment system on improvements in hospital infection management shows that systematic process monitoring facilitates continuous enhancement of training effectiveness, with a significant downward trend in the incidence of HAI post-implementation (31). Qualitative research on the absence of in-hospital infection control nursing measures and healthcare-associated infections has revealed that training process monitoring should also include tracking of trainees’ learning progress, assessment of the educational quality of trainers, and evaluation of training environments and resource allocation (32).

#### 4.3.3 Evaluation indicators for training effectiveness

The scientific establishment of indicators for evaluating training effectiveness is crucial for determining the success of training programs. It necessitates the creation of a multi-tiered and multidimensional evaluation system. The construction of a quality control indicator system for neonatal specialty hospital infection management provides a valuable reference for assessing training effectiveness. This system comprises three primary indicators, six secondary indicators, and 16 tertiary indicators, of which 13 can be directly sourced from the hospital information systems (33). Surveys on the current state of hospital infection management in non-public medical institutions reveal challenges in funding allocation, staffing, and the implementation of infection control measures. The most critical support needed is in the training of dedicated and part-time infection control personnel and in facilitating their professional advancement (34). This highlights that the evaluation of training effectiveness should not only focus on short-term improvements in knowledge and skills but also consider long-term professional development and capacity building. The exploratory practices in infection management across multiple hospital campuses indicate that the evaluation of training effectiveness must adapt to the unique characteristics and needs of different campuses. Employing comprehensive planning, homogeneous cultivation, and shared data management are essential to ensure the consistency and effectiveness of the evaluations (35).

## 5 Challenges and developmental trends in training for infection control liaison nurses

### 5.1 Predominant challenges in current training programs

#### 5.1.1 Imperfections in the training model

The training of infection control liaison nurses, while rapidly evolving, continues to face numerous challenges and issues. An analysis of multidrug-resistant bacterial infections and the efficacy of prevention controls at a tertiary hospital demonstrated that, despite a notable reduction in infection rates due to the implementation of a hospital infection management system, hospital-acquired infections still occurred in 12.62% of patients. This indicates that the sustainability and depth of training effects require further enhancement (36). Studies on the prevention of catheter-associated urinary tract infections using “bladder care packs” and the role of “infection control nurses” have identified the incomplete nature of the training model as a primary issue, particularly the lack of systematicity in training content, diversity in training methodologies, and the allocation of training resources (37). A survey on the use of protective gloves in routine disinfection of patient contact areas revealed that non-compliance with staffing requirements also significantly impacts the effectiveness of training. The survey showed clear discrepancies in job competency between full-time and part-time infection control personnel, with full-time staff consistently scoring higher across various dimensions.

#### 5.1.2 Lack of comprehensiveness in training content

Training content for ICLNs must be determined based on a thorough assessment of personnel demographics, existing job competency levels, and specific role requirements. Infection control personnel exhibit significant demographic characteristics, with over 90% holding a bachelor’s degree or less; additionally, there is a relative deficiency in training capability, research, and evidence-based skills. Training needs are primarily concentrated in four core areas: professional competence, comprehensive ability, professionalism, and personal traits. Therefore, the design of training content must be targeted, ensuring complete coverage of professional knowledge and skills in infection control on one hand, and on the other, providing stratified training for key demographics such as nurses with less experience and those with lower academic qualifications. Designing courses to address deficits in research, evidence-based practice, and management skills, and progressively advancing from basic to complex knowledge, is likely to achieve more ideal training outcomes.

### 5.2 Optimization strategy for training program

**Figure 1:**
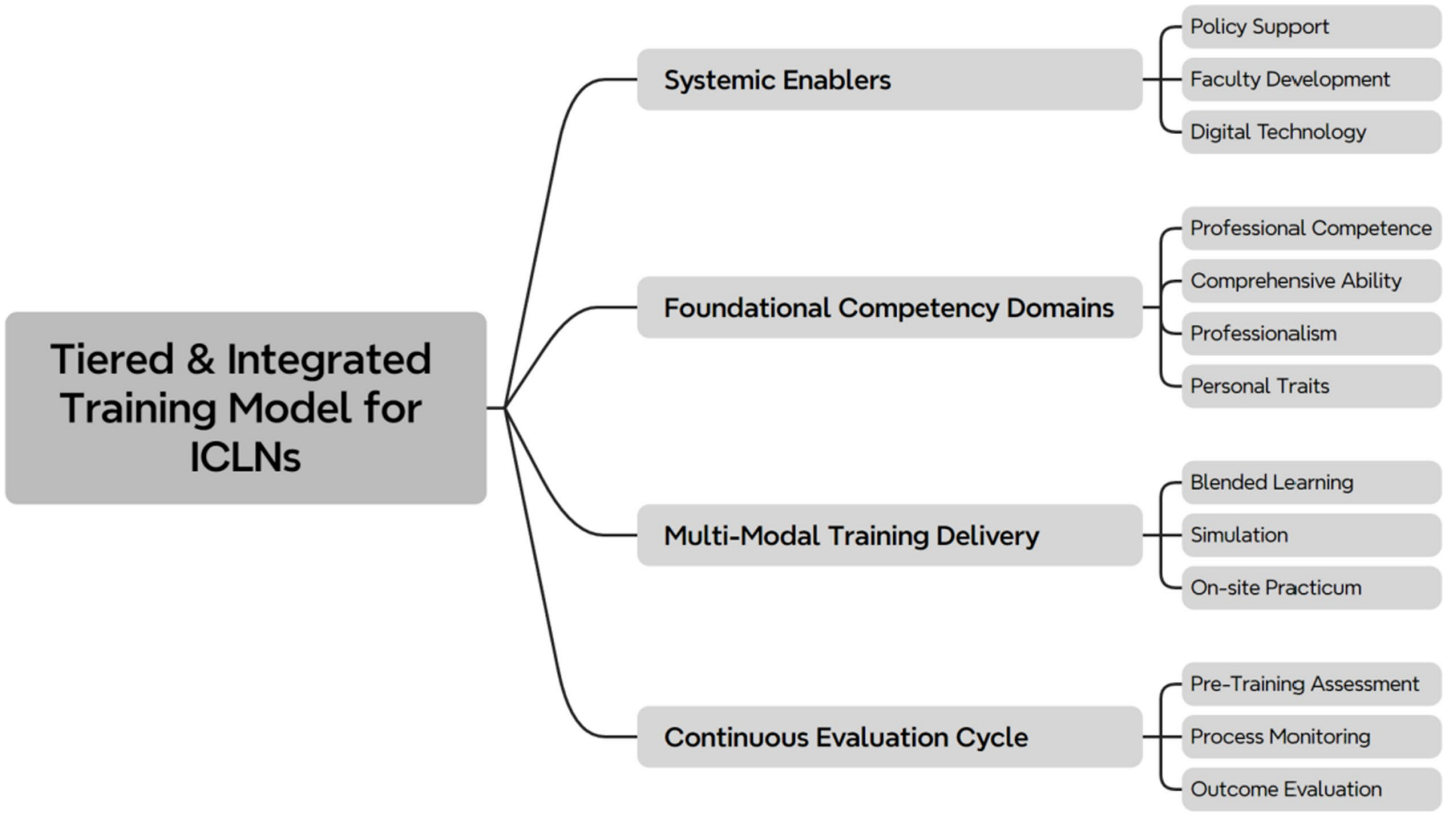
Proposed conceptual framework for a tiered and integrated training model for infection control liaison nurses.

In response to the challenges identified within training programs, it is essential to devise optimization strategies from multiple dimensions to enhance the pertinence and effectiveness of the training, as illustrated in Table 2. The core direction for optimization involves the establishment of a tiered and hierarchical training model. This system should be designed to accommodate the varying levels, sizes, specialties, and the roles, work experience, and professional backgrounds of infection control personnel within different medical institutions. A critical element in upgrading the quality of training involves strengthening the construction of the training faculty. This requires the development of a cadre of instructors who are not only profoundly theoretical but also possess extensive practical experience. Simultaneously, establishing a robust training and certification mechanism for instructors is imperative. The perfection of the training content system should focus on integrating theory with practice, encompassing the fundamental theoretical knowledge of infection control while emphasizing training in practical operational skills (see Figure 1). This includes the expansion of practice-oriented content such as case analyses, scenario simulations, and onsite drills (38).

**Table 2:** Framework for optimizing the training program for infection control liaison nurses (ICLNs).

Optimization dimensions	Main strategies	Specific measures	Expected outcome
System construction	Tiered and hierarchical training	Design differentiated plans based on hospital level and department characteristics	Improve training pertinence
Training faculty	Professionalization	Establish training and certification mechanisms for instructors	Ensure training quality
Content design	Theory-practice integration	Include case analyses, scenario simulations	Enhance practical application skills
Methodological innovation	Blend of online and offline modalities	Utilize digital platforms for diversified teaching	Improve training flexibility
Evaluation and feedback	Continuous improvement	Establish a sustainable evaluation and feedback mechanism	Ensure training effectiveness
Standardization	Unified certification	Develop training standards and certification systems	Ensure quality consistency

The framework integrates four core components: (1) Foundational Competency Domains (Professional Competence, Comprehensive Ability, Professionalism, Personal Traits); (2) Multi-Modal Training Delivery (Blended Learning, Simulation, On-site Practicum); (3) Continuous Evaluation Cycle (Pre-Training Assessment, Process Monitoring, Outcome Evaluation); and (4) Systemic Enablers (Policy Support, Faculty Development, Digital Technology). This model emphasizes the need for a structured yet flexible approach to address the varied needs of ICLNs across different healthcare settings.

### 5.3 Development trends and prospects

The evolution of the training program for infection control liaison nurses shows a trend towards specialization, precision, and intelligence. Specialization is manifested in the expanding depth and breadth of the training content, moving from foundational knowledge in infection control to more specialized and personalized directions. This is particularly evident in the growing training needs in specialized fields such as emerging infectious disease prevention, management of multidrug-resistant organisms, and prevention of infections related to medical devices. The competency evaluation indicator system, constructed using the Analytic Hierarchy Process-Entropy method, comprises four primary indicators, 15 secondary indicators, and 64 tertiary indicators. This system lays a crucial foundation for the scientific design of the training program, reflecting the systematic and scientific nature of training assessment (39). The precision in development is demonstrated through the design of the training program which increasingly focuses on individual differences and job-specific characteristics. It employs precise identification of training needs, enabling the formulation of personalized training pathways and learning plans (40). The intelligence dimension leverages new technologies such as big data and artificial intelligence to optimize the allocation of training resources, monitor the learning process intelligently, and precisely evaluate the effectiveness of the training.

## 6 Summary

The current analysis of the training needs and programs for infection control liaison nurses indicates that while significant progress has been made in this field in China, numerous challenges remain. The overall job competency of infection control personnel is at a moderately high level, yet there are significant disparities across different regions and levels of medical institutions. The training needs are increasingly diverse and personalized. While the construction of the training model is gradually improving and training methods are continuously being innovated, there is still room for enhancement in areas such as systematicity, pertinence, and sustainability. For instance, studies by She et al. (7) and Jiang et al. (6) consistently reported moderate-to-high competency scores among ICLNs, yet highlighted significant gaps in research and evidence-based skills. These findings, coupled with the identified weights in the competency evaluation index system (39), underscore the need for targeted training in these areas to enhance overall job performance. Future efforts should aim to construct a more scientific and comprehensive training model that emphasizes the deep integration of theory and practice, strengthens the development of the training faculty, innovates training methodologies, and refines the evaluation and feedback mechanisms. These improvements are essential to adapt to changes in the medical environment and the evolving needs of infection control work. Only through persistent efforts and improvements can we train more highly-qualified infection control liaison nurses, thereby ensuring patient safety, enhancing the quality of medical care, and contributing effectively to the prevention and control of hospital infections.

### 6.1 Limitations

This review has several limitations. First, the reliance predominantly on Chinese literature may limit the generalizability of findings to other healthcare systems and contexts. Second, as a narrative review, it did not include a systematic quality assessment or meta-analysis of the included studies, which may introduce selection bias and affect the strength of the conclusions. Third, the variability in study designs, measurement tools, and outcome reporting across the included literature complicates direct comparison and synthesis. Future research should incorporate more international studies and adopt systematic review or scoping review methodologies to enhance comprehensiveness and rigor.
